# Use of Seasonal Influenza Virus Titer and Respiratory Symptom Score to Estimate Effective Human Contact Rates

**DOI:** 10.2188/jea.JE20110146

**Published:** 2012-07-05

**Authors:** Szu-Chieh Chen, Shu-Han You, Min-Pei Ling, Chia-Pin Chio, Chung-Min Liao

**Affiliations:** 1 Department of Public Health, Chung Shan Medical University, Taichung, Taiwan, ROC; 2 Department of Family and Community Medicine, Chung Shan Medical University Hospital, Taichung, Taiwan, ROC; 3 Department of Bioenvironmental Systems Engineering, National Taiwan University, Taipei, Taiwan, ROC; 4 Department of Health Risk Management, China Medical University, Taichung, Taiwan, ROC

**Keywords:** influenza, behavior, contact rate, symptom score, modeling

## Abstract

**Background:**

We linked viral titers and respiratory symptom scores for seasonal influenza to estimate the effective contact rate among schoolchildren.

**Methods:**

We analyzed 274 diary-based questionnaires. In addition, 2 sets of influenza data from published studies were used to investigate the relationship between viral titer, total symptom score, and normalized contact rate in children.

**Results:**

The mean number (SD) of contacts for children in grades 7 to 9 ranged from 9.44 ± 8.68 to 11.18 ± 7.98 person^−1^ day^−1^; contact behavior was similar across school grades. The mean number of contacts was 5.66 ± 6.23 person^−1^ day^−1^ (range, 0 to 44 person^−1^ day^−1^) for the age group of 13 to 19 years. Estimated contact age, household size, contact duration, and contact frequency were the variables most strongly associated with total number of contacts. We also found that a reduction in total respiratory symptom scores among infected individuals had a positive correlation with an increase in the normalized contact rate.

**Conclusions:**

The relationship between daily virus titer and respiratory symptom score can be used to estimate the effective contact rate in explaining the spread of an airborne transmissible disease. The present findings can be incorporated into population-dynamic models of influenza transmission among schoolchildren.

## INTRODUCTION

Influenza is one of the most important infectious diseases affecting humans. The continued threat of human influenza pandemics provides the impetus to conduct long-term year-round surveillance of individual human influenza subtypes to improve our understanding of the disease.^1^ Most transmission of influenza probably happens within 3 feet of an infected person.^2^^,^^3^ Thus, the manner in which people interact within their social contact networks is critical to the spread of influenza infection. The most important parameters of transmission are the effective contact rate and the probability of transmission for a given social contact.^4^

Research on effective contact rates has involved investigating social mixing patterns relevant to infectious diseases and the development of transmission models.^5^^–^^12^ Different investigative methods and questionnaire tools have also been discussed and compared, including self-evaluation and diary-based data collection through a web-based interface^8^ and the use of hand-held electronic diaries (PDAs).^12^ In a pilot study of contact rate data, McCaw et al^12^ found that diary-based questionnaires were more acceptable than PDAs to participants. The timeliness, accuracy, and completeness of contact investigation are key points in estimating social contact.

Contact patterns are highly associated with age, and high rates of influenza transmission are particularly evident among school-aged children and teenagers. Thus, it is important that age-specific transmission parameters for respiratory infectious agents are estimated.^9^^,^^13^ Wallinga et al^9^ suggested that school-aged children and young adults have the highest incidence of infection and contributed most to the continued spread of infections during the initial phase of an emerging respiratory-spread epidemic among a completely susceptible population. Age groups investigated for contact included adults and young adults, elementary school students, and children younger than 11 years.^14^^,^^15^ Until now, the main focus of relevant research has been on the number of household contacts per day, due to the fact that the home is the principal intersection between the various locations frequented in our lives (such as schools and workplaces).^16^

Mapping social contact behavior to viral load is an interesting approach. Handel et al^17^ suggested that a sick person might reduce the frequency of contacts with others, that is, an increasing symptom score might be associated with behavioral changes. However, it is rarely possible to quantify the correlation between viral load and the social contact behavior of infectious individuals, primarily because most researchers can only capture the behavior of a susceptible individual while that person is healthy, and not after the individual’s behavior is potentially altered due to infection.

Much experimental research on human influenza A virus infection has studied local and systemic cytokine responses during the period of infection,^18^ as well as evidence for the safety and efficacy of oral and intravenous neuraminidase inhibitors.^19^^–^^22^ Different experimental trials have assessed dose, drug form, and participant responses (including daily viral titer, virus shedding, days of shedding, and clinical symptom scores). This research could assist in quantifying the inner viral titer and respiratory symptom score profile and in detailing systemic, upper respiratory, and lower respiratory symptoms.^19^^–^^22^

On the basis of the abovementioned concepts, this study used response surface analysis to investigate daily viral titers, daily TSSs, and daily normalized contact rates in schoolchildren. Specifically, the objectives of this study were to conduct a questionnaire-based survey to estimate effective contact rates and risk factors among junior high school students in March 2010 in Taiwan, quantify the dose (virus titer)–response (respiratory symptom score) relationship among infected volunteers, and combine this with the effective contact rate in children. We believe that this framework could be incorporated into analyses of transmission rates and in population-dynamic models used to develop control measures.

## METHODS

### Sample and questionnaire design

**Figure 1.**
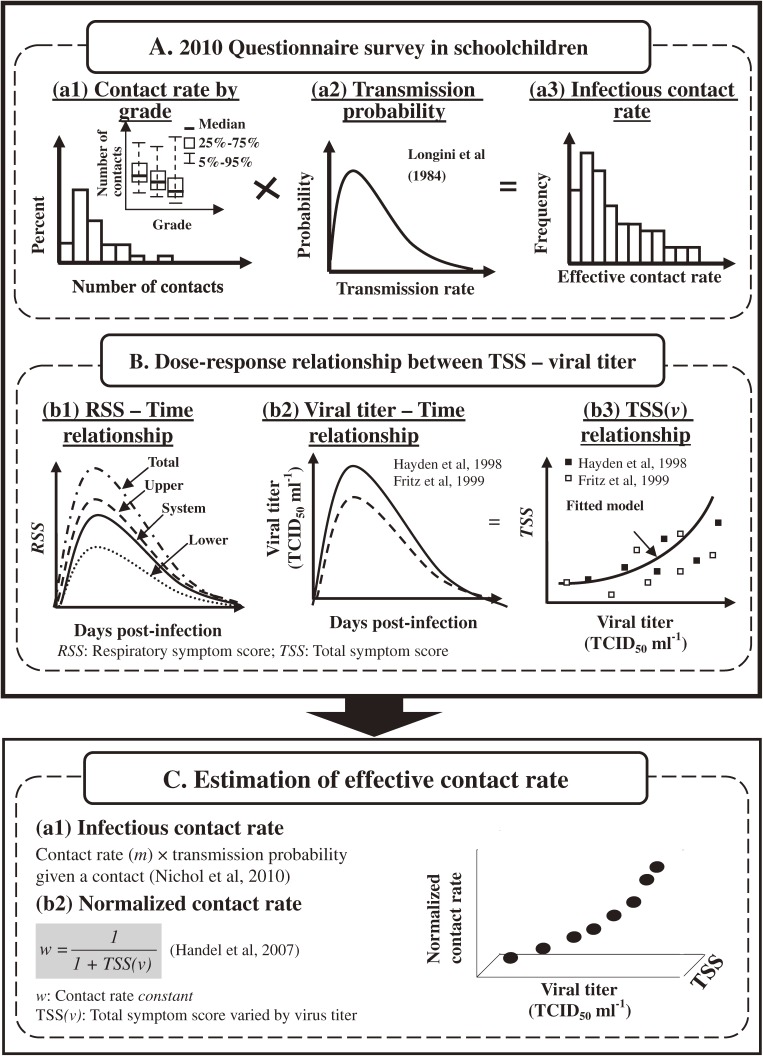
Research flowchart and study algorithm.

Figure 1 shows the research flowchart for the study. The questionnaire survey was approved by the Institutional Review Board of the Ethical Committees of Chung Shan Medical University. This study was conducted in a junior high school in Jhongli City, Taiyuan County, Taiwan, in March 2010. Junior high schools in Taiwan consist of grades 7 through 9 (age 12–14 years). The written informed consent of a parent or legal guardian was required and received, and older children were asked to give their assent.

The selected school was one of the larger junior high schools in Jhongli City, with 2861 students. There were 28 classes in each grade, and the average number of students in each class was 34. Two classes in each grade were selected to report their contacts on different days of the week. Each participant was asked to complete 2 questionnaires: 1 during a randomly assigned weekday and 1 during a randomly assigned day on the weekend. The participants were told in advance which days they had been assigned and were encouraged to complete the questionnaire before they went to bed.

The questionnaire (Appendix 1, see SUPPORTING INFORMATION) was developed in the form of a diary used to record all contacts during 1 day. The diary followed the course of the day divided into activities, starting with activities in the morning after awakening, on the way to school, play during breaks, and other activities after school until bedtime. A contact was defined as a 2-way conversation (at a distance that did not require raised voices) in which at least 3 words were spoken by each party, and in which there was no physical barrier between the 2 parties (such as a security screen).^5^ In addition, the conversation distance had to be less than 1 meter.^27^^,^^28^

At the beginning of the questionnaire, participants were asked to provide information on sampling date, sex, age, household size, their health status on that day (healthy, coughing, runny nose, headache, sneezing, or fever), whether or not they wore a protective mask, frequency of wearing such a mask (always, often, seldom), how well they remembered that day’s contacts (very well, well, moderately well, not well, poorly), and their influenza vaccination record for the past 6 months. All students were asked to list all contacts that they had had during the day, as well as the contact location (home, school, after-school tutoring, other), estimated age of contacts (0–5, 6–12, 13–19, 20–39, 40–59, ≥60 years), health status of contact (healthy, coughing, runny nose, headache, sneezing, or fever), whether contacts were wearing a mask, contact duration (<5 min, 5–15 min, 15 min–1 hr, 1–4 hr, >4 hr), contact frequency (every day, 1–2 times a week, 1–2 times a month, <1 time a month, first time), and contact level (level 1, level 2). Physical contact was divided into 2 types: a 2-way conversation during which at least 3 words were spoken (level 1), and a contact that involved any kind of skin-to-skin contact (level 2).

Statistical analyses were performed using SAS Version 9.1.3 for Windows (SAS Institute Inc., Cary, NC, USA). Correlation analysis was used to account for all variance in total number of contacts per person per day. Analysis of parameter estimates was based on best-fit Poisson regression models for all variance.

### Assessment of effective contact rate (per person)

Nichol et al^4^ defined the concept of infectious contact rate as a function of social mixing patterns and transmission probabilities for a given social contact (in which the infectious contact rate equals the number of daily contacts multiplied by the transmission probability).^4^ To assess the frequency distribution of an effective contact rate per person in grades 7 through 9 based on questionnaire results from junior high school students, we assumed that the mean number of contacts per day per person and variance of the total number of contacts would have a lognormal distribution. On the other hand, transmission probabilities between infected and susceptible contacts were estimated to range from 0.025 to 0.087 (ie, the probability of successfully infecting a susceptible individual in 1 contact).^29^ To calculate the effective contact rate per person per day, it was assumed that transmission probabilities were normally distributed with a mean of 0.05 and a variance of 0.0004. Using this technique, we were able to derive the grade-varied frequency distribution of the effective contact rate per person based on the results of the questionnaire and the assumed transmission probabilities.

### Relationship of daily viral titer to total symptom score

To investigate the relationship between influenza viral titer and symptom score, we used 2 excellent published studies of individuals who were infected with influenza A/Texas/36/91 (H1N1). These studies were chosen because they used a similar method to assess symptoms.^18^^,^^21^ The test age groups were age 19 to 40 years^18^ and 19 to 33 years,^21^ and the sizes of the study subgroups were 19^18^ and 8^21^ volunteers, respectively. Daily viral titer (log TCID_50_ ml^−1^) was estimated by using published data at day 0 (time of inoculation with inoculation dose 10^5^ TCID_50_) to day 7.

Symptoms were assessed twice daily by the volunteers, using a 4-point scale (0 for absent and 3 for severe) for each sign of specific systemic, upper respiratory, and lower respiratory symptoms. Higher scores indicated greater severity of symptoms. The individual symptoms that contributed to the TSS were divided into 3 subgroups: systemic signs (muscle aches, fatigue, headache, and fever), upper respiratory signs (nasal stuffiness, earache/pressure, runny nose, sore throat, and sneezing), and lower respiratory signs (cough, breathing difficulty, hoarseness, and chest discomfort). Daily TSSs were obtained by averaging the 2 symptom scores derived from Hayden et al^18^ and Fritz et al^21^ for that particular day. To investigate the representative dose–response relationship of infected volunteers, TSS was expressed as a function of the volunteers’ nasal influenza virus titer by fitting a function to data from Hayden et al^18^ and Fritz et al.^21^ Symptom score dataset analyses were performed using Didger 4 software (version 4.2, Golden Software, Inc.; Golden, CO, USA). TableCurve 2D software (Version 5.01, SYSTAT Software Inc.; Richmond, CA, USA) was used for curve fitting.

### Relationship between viral titer, total symptom score, and normalized contact rate in children

We used datasets from Cowling et al^25^ to investigate the relationship between viral titer, TSS, and normalized contact rate among children^25^ (Appendix 2, see SUPPORTING INFORMATION). Cowling et al^25^ provided viral titer data based on RT-PCR assays and cultures throughout the course of illness with pandemic and seasonal influenza among adolescents (age <16 years). Using the corresponding age groups in the questionnaire design, we compared the daily viral titer of children with the daily viral titer–symptom score relationship, which allowed us to estimate daily TSS among the children. The dataset was analyzed with Didger 4 software.

## RESULTS

### Description of sample characteristics

We collected a total of 404 questionnaires that covered all contacts made by junior high school students during a full day (Table 1). We excluded 130 of the returned questionnaires because they were incomplete, yielding a final effective sample size of 274 questionnaires (response rate, 67%). Table 1 shows numbers and percentages of participants across all groups, as well as mean number of contacts per person per day. The results show that the mean (SD) number of contacts for grades 7 through 9 ranged from 9.44 ± 8.68 to 11.18 ± 7.98 person^−1^ day^−1^, with similar contact behavior between school grades. Regarding number of members in a household, the largest number of survey participants were from households of 4 members (*n* = 65, 47.45%), and they accounted for almost 90% of the total sample. Each participant was asked to complete 2 questionnaires: 1 on a randomly assigned weekday and 1 on a randomly assigned weekend day. Fewer participants completed the survey on the weekday (23–34, 16.79%–24.82%) than on the weekend (62–75 participants, 45.26%–54.74%). Of the 274 total participants, 225 (82.12%) were healthy and 49 (17.88%) had at least 1 symptom when completing the questionnaires.

**Table 1.** Characteristics of participants and questionnaire responses (mean ± SD)^a^

	No. of participants (%)	No. of contacts (person^−1^ day^−1^)^a^
Sex		
Male	65 (47.45)	10.35 (8.59)
Female	72 (52.55)	9.99 (7.68)
School grade		
Grade 7	41 (29.93)	11.18 (7.98)
Grade 8	44 (32.12)	10.03 (7.54)
Grade 9	52 (37.96)	9.44 (8.68)
Household size		
2	1 (0.73)	4.5 (NA)^b^
3	13 (9.49)	7.35 (5.66)
4	65 (47.45)	11.15 (8.93)
5	29 (21.17)	10.94 (6.92)
>5	29 (21.17)	9.07 (8.1)
Day of the week		
Monday	23 (16.79)	11.70 (9.49)
Tuesday	27 (19.71)	10.63 (7.00)
Wednesday	26 (18.98)	11.65 (9.05)
Thursday	34 (24.82)	13.32 (6.85)
Friday	27 (19.71)	12.82 (10.14)
Saturday	62 (45.26)	8.46 (10.41)
Sunday	75 (54.74)	8.20 (5.49)
Health status^c^		
Healthy	225 (82.12)	10.55 (8.62)
1 symptom	30 (10.95)	7.10 (3.66)
2 symptoms	16 (5.84)	11.44 (5.72)
≥3 symptoms	3 (1.09)	4.67 (2.89)

^a^Total sample size of questionnaire = 404; effective sample size = 274: 39 questionnaires were not returned, 49 had incomplete data (missing sampling date or basic individual information) and 42 questionnaires had information for only 1 of 2 required days.

^b^Not available.

^c^Healthy, coughing, runny nose, headache, sneezing, and fever.

**Figure 2.**
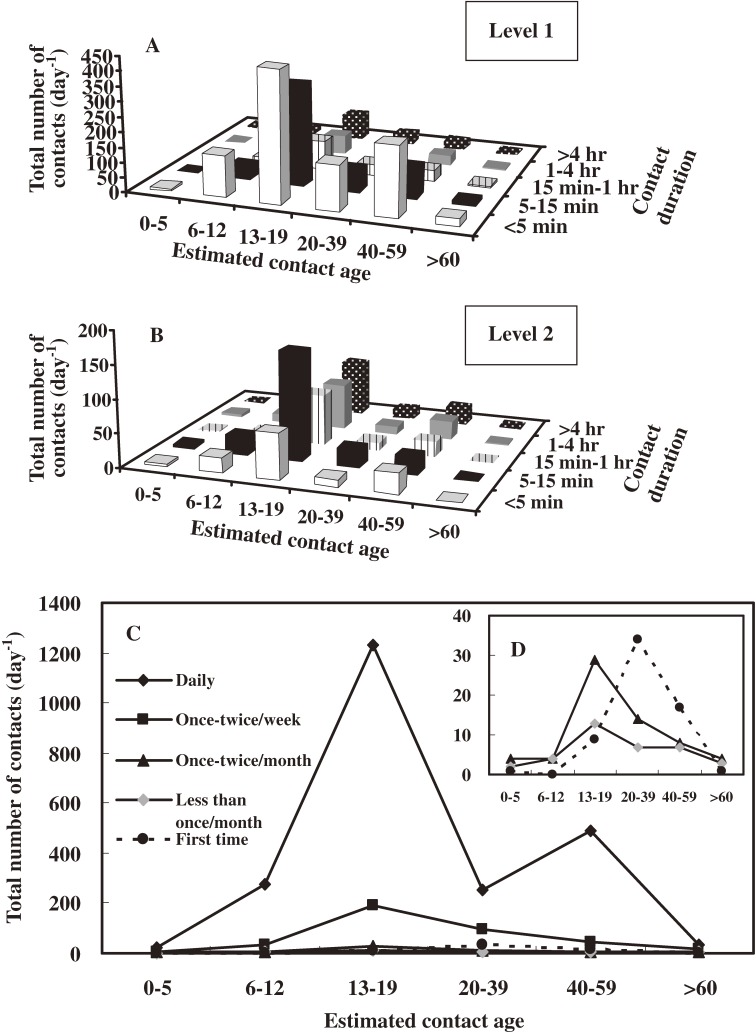
Analysis of data from the questionnaire survey. Total number of contacts (per day) by estimated contact age (ie, 0–5, 6–12, 13–19, 20–39, 40–59, ≥60 years) and variation in contact duration for (A) level 1 and (B) level 2 contacts. (C–D) Contact frequencies (daily, 1–2 times a week, 1–2 times a month, <1 time a month, first time) by total number of contacts (per day) are shown for the 5 age groups (0–5, 6–12, 13–19, 20–39, 40–59, ≥60 years).

Figures 2A and 2B show the total number of contacts per day for the 5 estimated contact age groups (0–5, 6–12, 13–19, 20–39, 40–59, ≥60 years) with regard to contact duration, contact frequency, and level of contact (level 1 or 2). Regardless of contact level, contact age groups of 13 to 19 years and 40 to 59 years had the highest total numbers of contacts among all contact age groups (250–400 contacts per day).

Regarding level 1 contacts (2-way conversations during which at least 3 words were spoken), a contact duration of less than 5 minutes was by far the most common duration in all age groups. For the estimated contact age group of 13 to 19 years, the results showed that, for level 1 contacts and a contact duration of less than 5 minutes (Figure 2A), 72% of contacts were at school and 12% at home, with 79% on a weekday and 21% on a weekend. In contrast, for the contact age group of 40 to 59 years, 84% of contacts were at home and 11% at other places, with 33% on a weekday and 67% on a weekend. It was most common for junior high school students to have contact with their classmates in school and their parents at home. In contrast, for level 2 contacts (contacts involving any skin-to-skin contact), contact duration was usually 5 to 15 minutes. In addition, for level 2 contacts and a contact duration of 5 to 15 minutes (Figure 2B) within the contact age group of 13 to 19 years, 73% of contacts were at school and 11% at home, with 80% on a weekday and 20% on a weekend. For the contact age group of 40 to 59 years, however, 70% of contacts were at home and 22.5% at other places, with 32% on a weekday and 68% on a weekend.

Figures 2C and 2D show the contact frequencies across the 5 estimated contact age groups for all contacts among school students. The results indicate that daily contact was recorded for all contacts, especially for the contact age groups of 13 to 19 and 40 to 59 years. Analysis of questionnaires indicated that the mean number of contacts was 5.66 ± 6.23 person^−1^ day^−1^ (range, 0 to 44 person^−1^ day^−1^) in the age group of 13 to 19 years. The results showed that contacts sufficient for transmission of infection were highly structured according to age (Figures 2C and 2D). In the age group of 40 to 59 years, the mean number of contacts was 1.96 ± 2.76 person^−1^ day^−1^ (range, 0 to 29 person^−1^ day^−1^).

### Statistical analysis and estimation of effective contact rate (per person)

**Figure 3.**
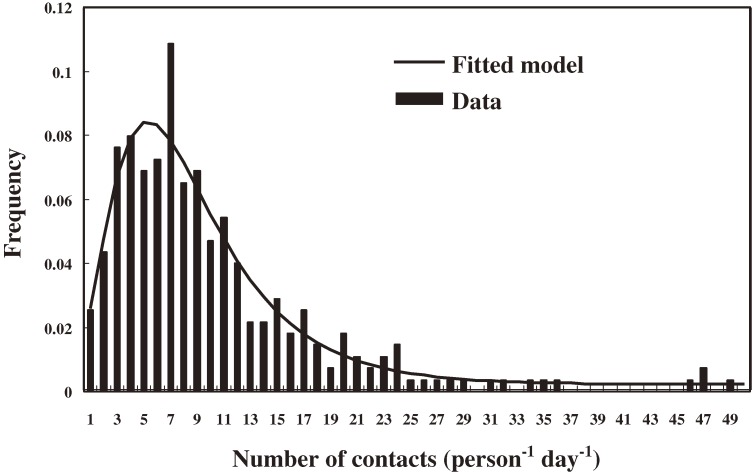
Frequency distribution of number of contacts as reported on questionnaires distributed in a junior high school in Jhongli City, Taiyuan County, Taiwan in March 2010. The histogram of the contacts and fitting curve show the survey data and optimal distribution, respectively. There were 274 effective sample sizes with 2804 recorded contacts (range, 0–49 per day).

**Figure 4.**
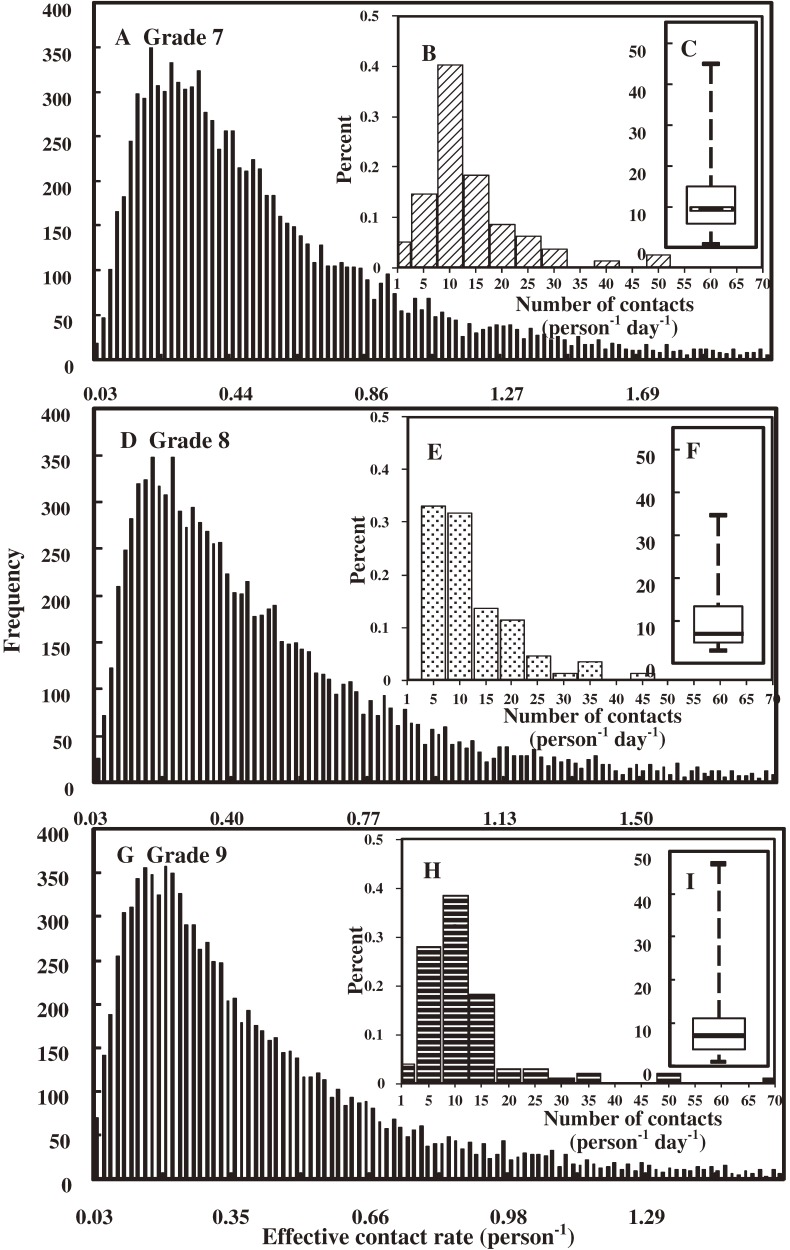
Effective contact rates for grades 7–9. The process was divided into 3 steps. First, the frequency distribution of the number of contacts (B, E, and H) for grades 7–9 was quantified by using the questionnaire data. The scale of the *x*-axis is subdivided into 15 subgroups (0–70 contacts), with an interval of 5 contacts. Second, box-whisker plots of the number of contacts were drawn and show the 5%–95% and 25%–75% percentile distributions for number of contacts (C, F, and I). Third, we calculated the frequencies of effective contact rates (A, D, and G) for grades 7–9 by multiplying the number of daily contacts by the transmission probability. A Monte Carlo simulation was used to analyze the probability distribution of transmission and number of daily contacts.

Figure 3 shows the frequency distribution of number of contacts (per person per day) at school. The results are strongly skewed and well fitted by the lognormal distribution (*R*^2^ = 0.94). The percentages in Figure 3 represent the number of participants with *x* number of contacts per day divided by the total number of participants. Figure 4 shows the effective contact rates (per person) based on the probability of transmission and mean number of contacts (per person). Figure 4 presents the frequency distributions of effective contact rate (per person) (Figures 4A, 4D, and 4G), frequency distributions of number of contacts (per person per day) (Figures 4B, 4E, and 4H), and box-whisker plots of the number of contacts (per person per day) (Figures 4C, 4F, and 4I) in the 3 grades. The results imply skewness in contact behavior, especially in the histograms for 5 to 10 and 10 to 15 contacts (Figures 4B, 4E, and 4H), with a best-fit lognormal distribution. The median effective contact rates for grades 7, 8, and 9 were 0.44, 0.38, and 0.31, respectively.

On the basis of findings from correlation analysis, several parameters were deleted from the Poisson regression models, including contact level, contact location, and symptom score, because they were not statistically significant (*P* > 0.05). Table 2 shows the analysis of parameter estimates based on best-fit Poisson regression models for all variances. Estimated contact age, household size, contact duration, and contact frequency were the most significant variables for total number of contacts per day.

**Table 2.** Analysis of parameter estimates based on best-fit Poisson regression models for all variances

Parameters	DF	Estimate	Standard error	Wald 95% confidence limits		Chi-square	Pr > ChiSq
Intercept	1	2.3355	0.0935	2.1522	2.5187	624.15	<0.0001
Estimated contact age	1	−0.0738	0.0185	−0.1100	−0.0376	15.98	<0.0001
Household size	1	0.0775	0.0271	0.0243	0.1307	8.15	0.0043
Contact duration	1	0.0796	0.0270	−0.1100	0.1326	8.66	0.0033
Contact frequency	1	0.1221	0.0330	0.0574	0.1869	13.67	0.0002

Abbreviation: DF, degrees of freedom.

### Relationship between virus titer and total symptom score

**Figure 5.**
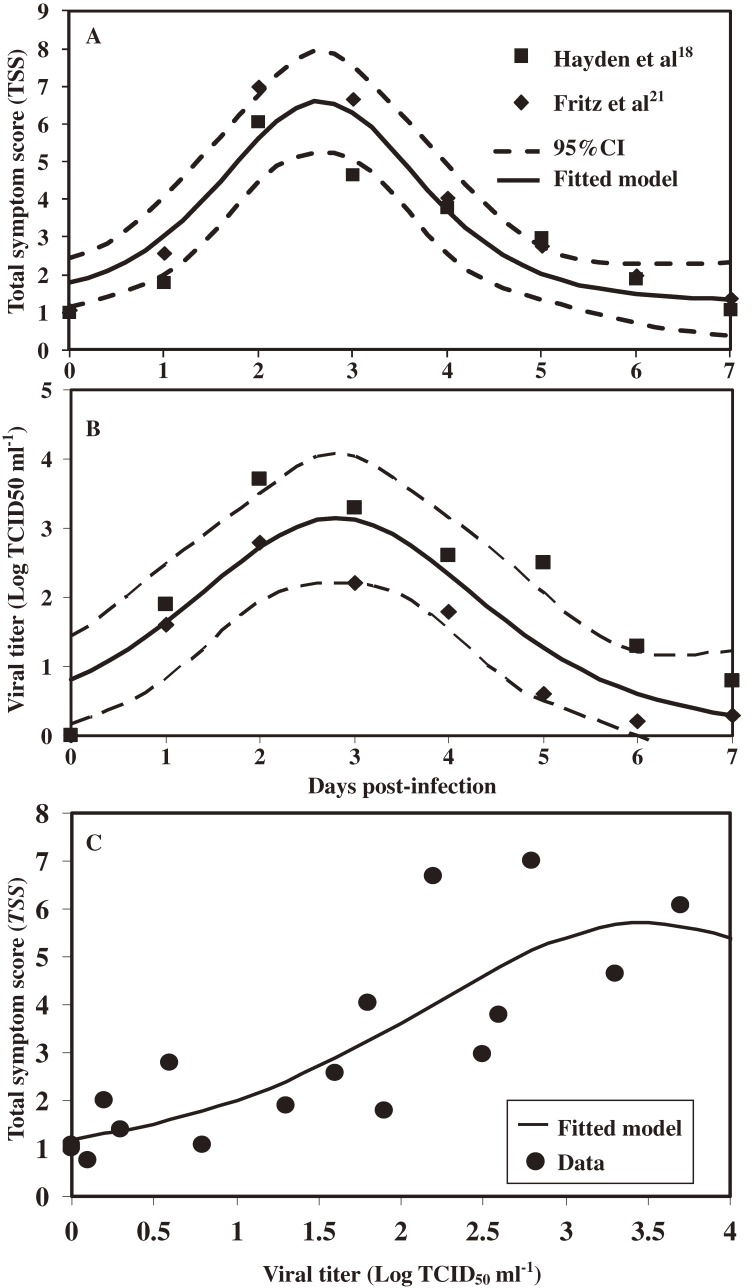
Data from peer-reviewed experimental human studies of the relationship between virus titer and total symptom score (TSS). (A) Time-dependent TSS by days post-infection, adopted from Hayden et al^18^ and Fritz et al.^21^ The time-dependent TSS can be expressed as: $TSS(t)=1.30+4\times \text{5.30}\times n/{\left(1+n\right)}^{2}$, n=exp(−(t−2.66)/0.71) with a logistic function curve (*R*^2^ = 0.84). (B) The time-dependent viral titer can be expressed as $v(t)=0.08+4\times \text{3.07}\times n/{\left(1+n\right)}^{2}$, n=exp(−(t−2.81)/1.04) with a logistic function curve (*R*^2^ = 0.70). (C) The best-fitted curve describing the relationship between virus titers and TSS.

Table 3 summarizes the data on experimental human influenza A (H1N1) infection and shows daily viral titers and symptom scores. TSS comprises upper and lower respiratory symptoms and systemic symptoms. Figure 5A shows time-dependent total respiratory symptom scores. Overall, the patterns show that symptoms started after day 1 and approached a peak score during days 2 and 3, then slowly decreased to less than 1 at day 7. Upper respiratory symptoms were more severe than systemic scores in both referenced studies (Table 3).^18^^,^^21^ We used daily viral titer (Figure 5B) combined with daily-based TSS (Figure 5A) to assess the relationship between virus titer and TSS with a best-fit logistic function (*R*^2^ = 0.67) (Figure 5C).

**Table 3.** Daily viral titers and symptom scores for upper respiratory, lower respiratory, and systemic symptoms

Days post-infection	Viral titer (*v*, LogTCID_50_ ml^−1^)	Symptom scores^a^			

		Upper respiratory symptom score^b^	Lower respiratory symptom score^c^	Systemic symptom score^d^	Total symptom score^e^
Hayden et al^18^					
0	0	0.62	0.06	0.32	1
1	1.9	1.13	0.21	0.45	1.79
2	3.7	3.18	0.47	2.41	6.06
3	3.3	2.94	0.47	1.24	4.65
4	2.6	2.57	0.45	0.76	3.78
5	2.5	1.52	0.62	0.84	2.98
6	1.3	0.9	0.47	0.52	1.89
7	0.8	0.65	0.32	0.1	1.07
Fritz et al^21^					
0	0	0.2	0.27	0.6	1.07
1	1.6	1.43	0.21	0.94	2.58
2	2.8	3.9	0.43	2.67	7
3	2.2	3.9	0.77	2	6.67
4	1.8	2.3	0.55	1.2	4.05
5	0.6	1.8	0.34	0.63	2.77
6	0.2	1.15	0.27	0.57	1.99
7	0.3	0.7	0.22	0.46	1.38

^a^Symptom assessments were conducted twice a day for volunteers, using a 4-point scale for specific symptoms (0–3 from absent to severe).^18^^,^^21^

^b^Nasal stuffiness, earache/pressure, runny nose, sore throat, hoarseness, and sneezing.

^c^Coughing, breathing difficulty, hoarseness, and chest discomfort.

^d^Muscle aches, fatigue, headache, and fever.

^e^Total symptom scores for upper respiratory, lower respiratory, and systemic symptoms.

### Contact rate, respiratory symptom score, and virus titer

**Figure 6.**
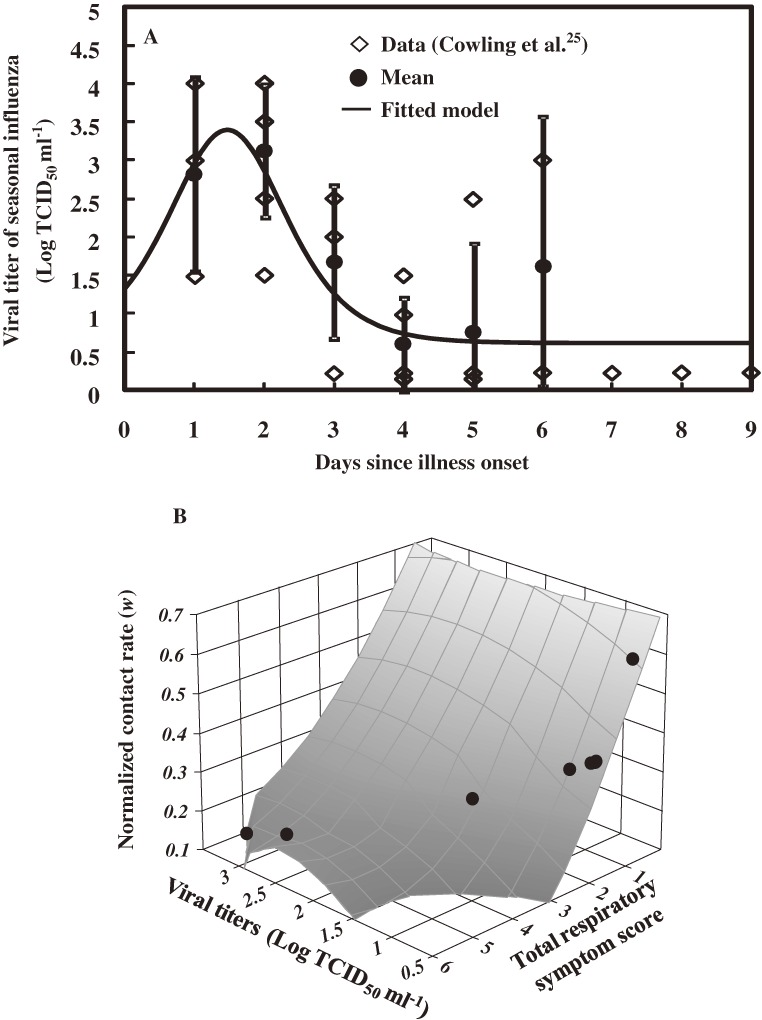
Relationship of contact rate, respiratory symptom score, and virus titers. (A) Viral titers of seasonal influenza at days since illness onset for adolescent (age, <16 years) participants.^25^ (B) The response surface shows the relationship between daily viral titer, TSS, and normalized contact rate among children.

Table 4 summarizes the assessment process for daily viral titer, TSS, and normalized contact rate for children. The estimated time-dependent viral titer, *v*(*t*), of children can be expressed as

$$\begin{array}{l} v(t)=0.62+4\times 0.278\times n/{\left(1+n\right)}^{2},\\ n=\exp(-(t-1.61)/0.61), \end{array}$$

with a logistic function curve (*R*^2^ = 0.60) (Figure 6A ).

**Table 4.** Relationship between daily viral titer, total symptom score, and normalized contact rate

Days since illness onset (*t*)	Viral titer (*v*(*t*), LogTCID_50_ ml^−1^)^a^	Total symptoms score^b^ (*TSS*(*t*))	Normalized contact rate^c^ (*w*(*t*))
1	2.81	5.14	0.16
2	3.13	5.55	0.15
3	1.57	2.82	0.26
4	0.84	1.83	0.35
5	0.66	1.65	0.38
6	0.63	1.62	0.38
7	0.62	1.62	0.38
8	0.62	0.66	0.60

^a^Viral titers for children were taken from Cowling et al.^25^ The time-dependent viral titers for children can be expressed as:

$$\begin{array}{l} v(t)=0.62+4\times 0.278\times n/{\left(1+n\right)}^{2},\\ n=\exp(-(t-1.61)/0.61)(\text{Figure 6A}). \end{array}$$

^b^The total symptom score for children was estimated by integrating the viral titer of children with the relationship between virus titer and symptom score. Hence, the time-dependent total symptom score of children can be expressed as:

$$\begin{array}{l} TSS(t)=0.66+4\times 0.505\times n/{\left(1+n\right)}^{2},\\ n=\exp(-(t-3.49)/0.97)(\text{Figure 5C}). \end{array}$$

^c^Estimated by the equation w(t)=1/(1+TSS(v)).

TSS was estimated based on viral titer among children (Figure 6A), and the relationship between viral titer and TSS was

$$\begin{array}{l} TSS(t)=0.66+4\times 0.505\times n/{\left(1+n\right)}^{2},\\ n=\exp(-(t-3.49)/0.97)(\text{Figure 5C}). \end{array}$$

On the basis of the findings of Handel et al,^17^ the normalized contact rate was found to range from 0.16 to 0.60 (Table 4).

The relationship between daily viral titer, TSS, and normalized contact rate is shown in Figure 6B. Normalized contact rate increased during the days after illness onset, while viral titer and TSS decreased accordingly. We observed that a decrease in the TSS for an infected individual could increase the normalized contact rate.

## DISCUSSION

To estimate effective contact rates and risk factors for influenza transmission, we conducted a questionnaire-based survey of Taiwanese junior high school students. We specifically investigated the relationship between dose (virus titer) and response (respiratory symptom scores) of infected volunteers and the effective contact rate among children.

We found that the mean numbers of contacts for children in grades 7, 8, and 9 were 11, 10 and 9, respectively, indicating similar contact behavior among junior high school students in different grades. The number of reported contacts varied significantly depending on estimated contact age, household size, and contact duration and frequency (estimated via Poisson regression models). Our findings for children who mixed mostly with others of similar age were consistent with those of previous studies.^7^^,^^11^^,^^13^^,^^14^ In addition, an association between household size and total number of reported contacts was confirmed.^12^^,^^13^ Contact duration and contact frequency were related because children had a fixed, regular lifestyle (ie, they travelled between school and home from Monday through Friday). Flu symptoms (1, 2, and ≥3 symptoms) were reported by 17.88% of participants. Among healthy participants, the mean number of contacts was 10.55 person^−1^ day^−1^ in grades 7 to 9. However, the survey questionnaire did not allow a full comparison of baseline contact rates between the healthy population and infected individuals.

This study defined contact as a 2-way conversation during which at least 3 words were spoken by each participant, there was no physical barrier between the 2 speakers, and the distance between speakers was less than 1 meter. However, this ignores other potentially important routes of transmission such as indirect contact (through inanimate objects contaminated with infectious agents), long-distance airborne transmission, and exposures that do not involve conversation or touch (a sneezing bus passenger, for example).

A total of 404 questionnaires were distributed, with a 67% response rate. Participants were classified as nonresponders when their questionnaire was unreturned (9.6%), incomplete (12.1%), or missing data for 1 weekday or 1 weekend day (10.3%). The proportion of nonresponders suggests that some participants did not pay attention to the purpose of the questionnaire or did not understand the importance of disease transmission and indicates a need to enhance the executive process for questionnaire investigation.

Edmunds et al^5^ used the WAIFW (who acquires infection from whom) matrix to assess patterns of contact within and between age groups indirectly^23^^,^^24^ and found that estimates of effective contact rates were derived from estimates of the instantaneous per-susceptible infection rate (force of infection) for each of the *n* age groups. Mikolajczyk et al^15^ used an equation by Anderson and May^24^ to estimate an effective contact rate (*C*) from the mean (*m*) and the variance (*v*) of the number of contacts in a heterogeneous population, expressed as C=m+v/m. A recent study by Nichol et al^4^ used the infectious contact rate to explain social mixing patterns and transmission probability per contact for the population. Using the same method as Nichol et al,^4^ we found median effective contact rates, (*C_E_*) (95% CI), of 0.44 (0.1–2.07), 0.38 (0.08–1.89), and 0.31 (0.06–1.59) for grades 7, 8, and 9, respectively, based on the number of contacts multiplied by the transmission probability.

There are only limited data on viral shedding patterns associated with naturally acquired influenza virus infections in teenagers. The viral load of seasonal influenza^25^ was used to estimate viral load in children (age, <16 years) in this study. On the basis of the findings of Handel et al^17^ the normalized contact rate as a function of viral load and respiratory symptom score was estimated to be 0.16 to 0.60. Using 2 different methods, this study arrived at similar values for the effective contact rate and normalized contact rate among junior high school students. Both methods indicated that the effective number of contacts for influenza transmission was less than 1 contact per day per person.

The relationship between daily viral titer, daily TSS, and daily normalized contact rate was also investigated. We found that a reduction in the total respiratory symptom score for an infected individual had a positive correlation with an increase in the normalized contact rate. This may be due to the mild symptoms of infected individuals who went outside and came into contact with other susceptible individuals.

An implication of this study is that rates of transmission between susceptible and infectious individuals can be quantified and integrated into dynamic population modeling of schoolchildren. Stilianakis and Drossinos^26^ found that transmission rates could be estimated by multiplying 2 figures: the contact rate of a susceptible individual to a droplet exhaled by an infectious individual, and the probability that contact with an exhaled droplet results in successful transmission to a susceptible individual. The present study quantified contact rate using survey questionnaires, and these data can be used to estimate transmission rates in the future.

In conclusion, this study is the first to integrate daily virus titer and respiratory symptom scores to estimate effective contact rate and explain the spread of an airborne transmissible disease. Seasonal influenza viral titer combined with respiratory symptom scores can be used to estimate effective contact rates and could potentially be integrated into dynamic population models of infectious diseases.

## SUPPORTING INFORMATION

Appendices are available on the journal's website at http://dx.doi.org/10.2188/jea.JE20110146.

eAppendix.
