# Supplementary material for: Use of Seasonal Influenza Virus Titer and Respiratory Symptom Score to Estimate Effective Human Contact Rates
Source: J Epidemiol. 2012 Jul 5;22(4):353–63. doi: 10.2188/jea.JE20110146 (PMC3798655; doi:10.2188/jea.JE20110146)
Supplement: eAppendix. [file je-22-353-s001.pdf]

Supplementary materials

Appendix 1: Questionnaires

Dear participants:

We are investigating the number and characteristics of daily contacts among schoolchildren. Please take a few minutes to complete the questionnaire carefully and answer the questions on the basis of your actual situation. The questionnaires will remain anonymous. Please give us your valuable comments, which will be used for research purposes only. All information you supply will be treated in strict confidence. We appreciate your help. If you have any questions about this questionnaire, please contact us.

Graduate student: Shu-Han You.
Phone number: +886-920-991-879; E-mail: emma80093@hotmail.com
Department of Public Health, Chung Shan Medical University, Taichung, Taiwan

Instructions

A. The conditions of a contact are as follows:
(*i*) A 2-way conservation (at a distance that did not require raised voices) in which at least 3 words were spoken by each speaker.
(*ii*) A distance of less than 1 meter between speakers.
(*iii*) No physical barrier (eg, a window, door, or glass) between speakers.

B. Please complete the questionnaire before you go to bed.

C. The period covered by the questionnaire starts with activities in the morning after awakening and includes the commute to school, play during breaks, and other activities after school.

D. Please refer to the second section, “Explanation of codes,” when you complete the third section, “List of contacts.”

1. Personal information

A. Date: ________

B. Sex: ☐Male ☐Female

C. Age: ________

D. How many people live with you (include yourself)? ☐2 ☐3 ☐4 ☐5 ☐>5

E. Family members who live with you:

☐Father ☐Mother ☐Brother____ ☐Sister____ ☐Younger brother____ ☐ Younger sister____

F. Where do you live now?

☐Home ☐Dormitory ☐Other______

G. How would you describe your health today (indicate all symptoms)?

☐Healthy ☐Coughing ☐Runny nose ☐Headache ☐Sneezing ☐Fever

H. Did you wear a protective mask today?

☐No ☐Yes (☐Always ☐Often ☐Seldom)

I. How well do you recall your contacts today?

☐Very well ☐Well ☐Moderately well ☐Not well ☐Poorly

J. Were you vaccinated for influenza during the past 6 months?

☐Yes, _____times ☐No

K. Did you have any problems or suggestions regarding the questionnaire?

____

____

2. Explanation of codes

**Option**	1.Contact place	2.Health status (Multiple choice)	3.Mask use	4.Age range (years)	5.Contact level	6.Contact duration	7.Contact frequency
**Codes**	(A)Home<br>(B)School<br>(C)After-school tutoring<br>(D)Other	(A)Healthy<br>(B)Coughing<br>(C)Runny nose<br>(D)Headache<br>(E)Sneezing<br>(F)Fever	(A)Yes<br>(B)No	(A)0–5<br>(B)6–12<br>(C)13–19<br>(D)20–39<br>(E)40–59<br>(F)≧60	(A)Talking<br>(B)Talking and touching	(A)<5 min<br>(B)5–15min<br>(C)15min–1hr<br>(D)1–4 hr<br>(E)>4 hr	(A)Everyday<br>(B)1–2 times a week<br>(C)1–2 times a month<br>(D)<1 time a month<br>(E)First time

3. List of contacts

Option	1. Contact place	2. Health status (Multiple choice)	3. Mask use	4. Age range (years)	5.Contact level	6.Contact duration	7.Contact frequency
1							
2							
3							
4							
5							
6							
7							
8							
9							
10							
11							
12							
13							
14							
15							
16							
17							
18							
19							
20							

Appendix 2: Method of mapping human behavior

Mapping human behavior based on viral load was described in the Appendix of Handel et al.[17] Their findings showed that a sick person might reduce their frequency of contact with other people, that is, a higher symptom score might be associated with behavioral changes. Handel et al[17] defined normalized contact rate (*w*) as a function of viral load (*v*), namely, $w(t) = 1/\left(1 + TSS\left(v\right)\right)$. On the basis of their ideas, we investigated the relationship between daily viral titer, daily total symptom score, and daily normalized contact rate. Table Curve 3D software (Version 4.0, 1993 – 2002; SYSTAT Software Inc., Richmond, CA, USA) was used for model fitting.
